# Changes in nanomechanical properties of single neuroblastoma cells as a model for oxygen and glucose deprivation (OGD)

**DOI:** 10.1038/s41598-022-20623-8

**Published:** 2022-09-29

**Authors:** Tomasz Zieliński, Joanna Pabijan, Bartłomiej Zapotoczny, Joanna Zemła, Julita Wesołowska, Joanna Pera, Małgorzata Lekka

**Affiliations:** 1 Department of Biophysical Microstructures, Institute of Nuclear Physics, Polish Academy of Sciences, PL-31342 Kraków, Poland; 2 Laboratory of in Vivo and in Vitro Imaging, Maj Institute of Pharmacology, Polish Academy of Sciences, Smętna 12, 31343 Kraków, Poland; 3 Department of Neurology, Faculty of Medicine, Jagiellonian University Medical College, Botaniczna 3, 31503 Kraków, Poland

**Keywords:** Nanoscience and technology, Nanomedicine, Biophysics, Nanoscale biophysics

## Abstract

Although complex, the biological processes underlying ischemic stroke are better known than those related to biomechanical alterations of single cells. Mechanisms of biomechanical changes and their relations to the molecular processes are crucial for understanding the function and dysfunction of the brain. In our study, we applied atomic force microscopy (AFM) to quantify the alterations in biomechanical properties in neuroblastoma SH-SY5Y cells subjected to oxygen and glucose deprivation (OGD) and reoxygenation (RO). Obtained results reveal several characteristics. Cell viability remained at the same level, regardless of the OGD and RO conditions, but, in parallel, the metabolic activity of cells decreased with OGD duration. 24 h RO did not recover the metabolic activity fully. Cells subjected to OGD appeared softer than control cells. Cell softening was strongly present in cells after 1 h of OGD and with longer OGD duration, and in RO conditions, cells recovered their mechanical properties. Changes in the nanomechanical properties of cells were attributed to the remodelling of actin filaments, which was related to cofilin-based regulation and impaired metabolic activity of cells. The presented study shows the importance of nanomechanics in research on ischemic-related pathological processes such as stroke.

## Introduction

Ischemic stroke remains one of the leading causes of death, especially in the elderly^1^. It is caused by disrupted blood flow to the brain resulting in oxygen and glucose deficiencies in the cells. The last three decades show significant improvements in acute treatment, resulting in increased life expectancy after treatment and rehabilitation^1,2^. Understanding the stroke at the cellular level can be simulated using an in vitro oxygen–glucose deprivation (OGD) model. The model was widely investigated to study ischemic cell death^3^. In the model, cells or tissue slices are exposed to hypoxic or anoxic conditions and cultured in glucose-deprived media. Not only the effect of OGD is investigated in the model—after changing media and introducing normal oxygen levels, reperfusion can be additionally tested. With long-lasting OGD, reoxygenation may paradoxically cause additional damage. Ischemia–reperfusion injury is caused by the immediate generation of reactive oxygen species, altered ion transport, and calcium influx^4^. During OGD, rapid remodeling of the actin cytoskeleton was reported to be involved in the blood–brain barrier disruption and affected: endothelial cells^5^, non-neuronal brain cells^6^, and neurons^7^.

Actin filaments occur in a cell as a meshwork or bundles of parallel fibers abundant, particularly close beneath the cell membrane^8,9^. The continuous control of the balance between polymerization and depolymerization ensures a dynamic equilibrium state, controlling cell architecture, mechanical resistance, and regulating many biological processes^10^. The dynamic of this process is regulated by actin-binding proteins^11^. Cofilin, an actin-depolymerizing factor, was highlighted several times to play a crucial role in actin remodeling in axons^7,12,13^. In ischemia-induced actin disruption, cofilin was linked with ATP depletion^14^. It has already been reported that cofilin is essential for an early phase of apoptosis^15^ or intracellular contractile force generation^16^. The responsibility of cofilin and its role in various diseases makes it a potential target for potential neuroprotective approaches in the early stages of ischemic brain injury. In particular, the SH-SY5Y human neuroblastoma cell line is used to investigate the OGD model of stroke^17^. The cell line is of human origin, allowing for a better reflection of the induced changes during the stroke. Both non-differentiated and differentiated SH-SY5Y cells have their advantages and drawbacks in the model of neuron cells^18^. In this report, we used undifferentiated cells, which are considered to be most reminiscent of immature neurons^19,20^. In the present study, we hypothesize that the involvement of cofilin occurs in the early stages of cytoskeleton remodelling under ischemic conditions. In the initial phase, it is limited to actin filament reorganization, which can be quantitatively evaluated using an atomic force microscope (AFM)^21^. This technique is characterized by nanoscale resolution that quantifies fine alterations in cells and tissue nanomechanical properties in normal and pathological conditons^22–25^.

Both undifferentiated and differentiated SH-SY5Y cells have been applied in "in vitro*"* studies that require neuronal-like cells^26,27^. Undifferentiated SH-SY5Y cells continuously proliferate, express immature neuronal markers, and lack mature neuronal markers^28^. Undifferentiated cells are considered to be most reminiscent of immature catecholaminergic neurons^29,30^. In our study, we decided to use SH-SY5Y cells because their OGD response is more prolonged than primary neurons, which enables us to elaborate the protocol of AFM measurements and relate the results to the cell cytoskeleton. As AFM can only measure cells that are attached to a surface, the early effects of OGD (before cell detachment) can be probed by this technique. The changes in mechanical properties have already been shown in undifferentiated SH-SY5Y cells in a model of chemically induced neurodegeneration^31^. The results, associated with glutamate-mediated neurodegeneration, showed the increased rigidity of SH-SY5Y cells upon 50 mM N-methyl-D-aspartate (NMDA) treatment, which indicates that NMDA induced cytoskeletal reorganization. Although the experiment time was limited to 60 min, the maximum rigidity values were obtained after 20 minutes^31^. However, knowledge about the mechanical changes in OGD/reoxygenation (RO) is still lacking.

In our work, we have focused on the undifferentiated SH-SY5Y cells because they are characterized by neuroblast-like, non-polarized cell morphology with few truncated neurite-like structures. Thus, we analyzed the nanomechanical properties of SH-SY5Y neuroblastoma cells exposed to oxygen and glucose deprivation, mimicking ischemic conditions. Following our previous studies on the effect of anti-tumor drugs on prostate cancer cells^32^, two indentations were applied, i.e., shallow (400 nm) and deep (1200 nm) ones. The shallow indentation reveals mechanical properties linked with actin filament organization, while the deep indentation may contain the additional contribution from deeper parts of the cells like the microtubular network and cell nucleus^32,33^. The studies were accompanied by evaluating the cofilin and phosphorylated cofilin expression levels, visualization of actin filaments organization quantified using morphometric parameters, and metabolic activity of SH-SY5Y cells subjected to OGD. Measurements were conducted directly after OGD to study the magnitude of the induced changes and after 24 h of reoxygenation to model reperfusion and to evaluate the reversibility of these changes.

## Results

### Viability of SH-SY5Y cells under OGD

**Figure 1:**
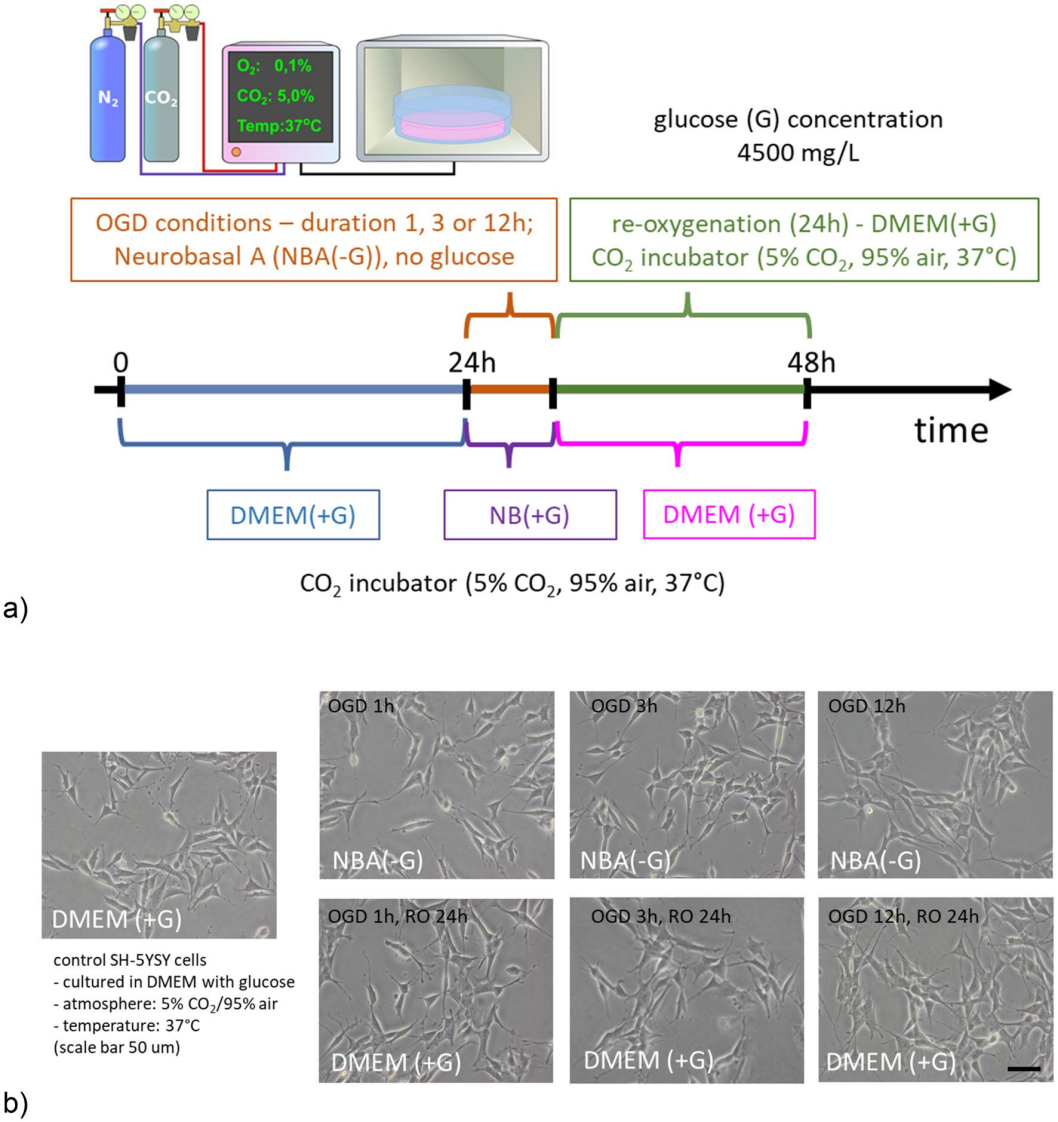
(**a**) A scheme showing three steps of sequential OGD applied to living SH-SY5Y cells. Firstly, cells were cultured for 24 h after seeding in 5% CO_2_, 95% atmosphere (37 °C) in a DMEM with 4500 mg/ml of glucose (DMEM(+ G)). They refer here as control cells. Next, the medium was exchanged to NBA(-G), and cells were placed in a table CO_2_ incubator for 1 h, 3 h, or 12 h at 0.1% O_2_ (referred to as OGD conditions and OGD cells). Finally, OGD cells were rinsed with a DMEM(+ G) in the atmosphere of 5% CO_2_ and 95% air (reoxygenation conditions, in addition, non-OGD cells were kept in DMEM(+ G)). **(b)** Phase-contrast image showing the morphology of neuroblastoma SH-SY5Y cells cultured for 24 h in NB(+ G), as it induced differentiation resulting in a neuron-like morphology with numerous, fine protrusions (neurite-like structures). Scale bar 50 µm.

To assess the effect of OGD exposure (5% CO_2_, 0.1% O_2_) on neuroblastoma SH-SY5Y cells, we exposed these cells to OGD for 1, 3, and 12 h, followed by 24 h-RO (Fig. 1).

Four groups of data were compared: (i) control (C, measurements were conducted in neurobasal medium, which contained 4500 mg/L of glucose, referred to here as NB(+ G)), (ii) OGD cells (in neurobasal A medium without glucose, NBA(-G)), (iii) re-oxygenated OGD cells (DMEM, which contained 4500 mg/L of glucose, DMEM(+ G)) and (iv) additional control (i.e., non-OGD) cells kept in DMEM(+ G) for the same time as re-oxygenated (RO) OGD cells.

**Figure 2:**
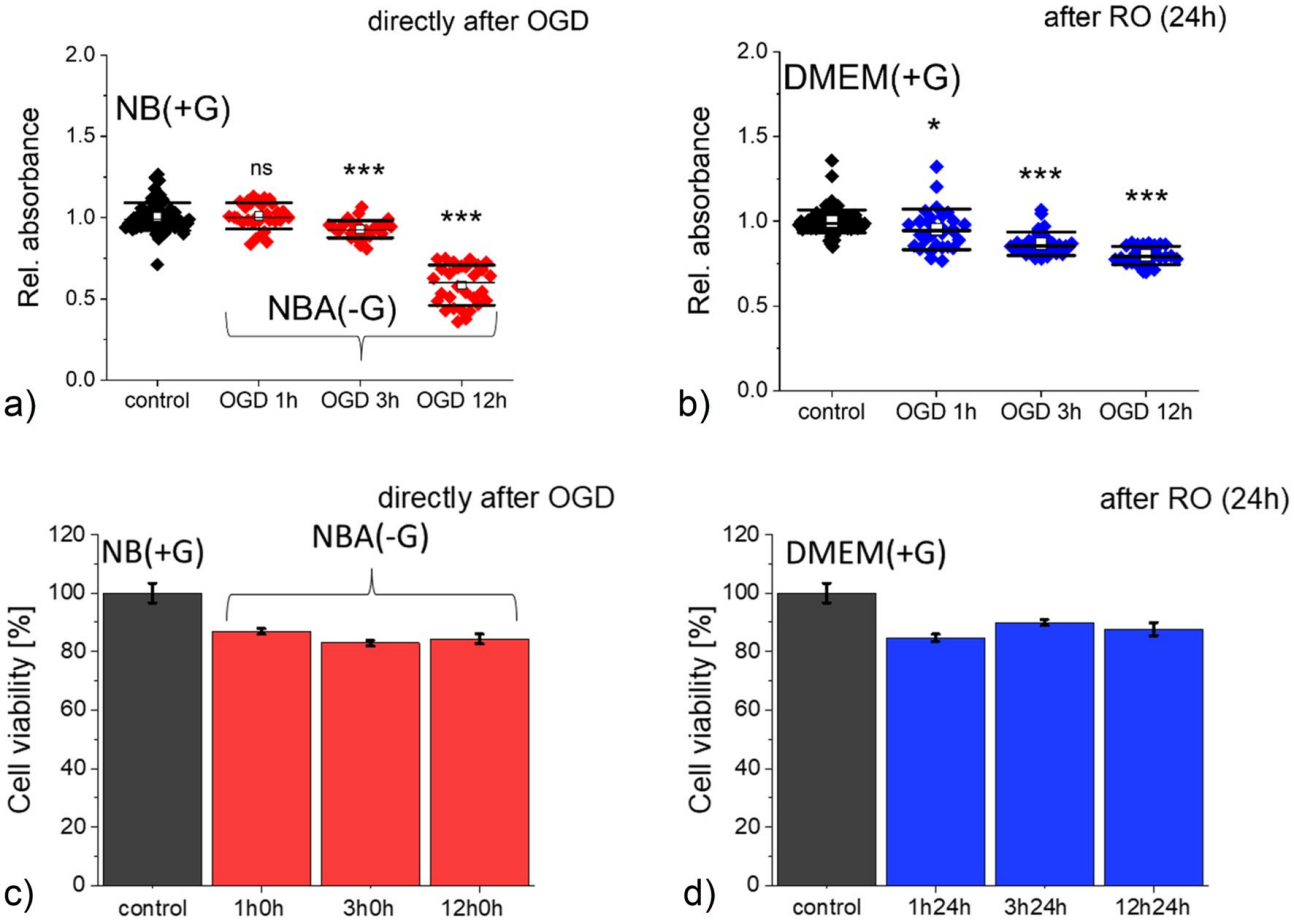
Metabolic level and viability of SH-SY5Y neuroblastoma cells assessed by MTS (**a,b**) and LDH (**c,d**) assays, directly after OGD (**a**,**c**) and after 24 h of reoxygenation (**b,d**). Each dot denotes a single readout from the ELISA reader. (**a,b**) A mean (open circle), median (black line), standard deviation (SD, box size) were determined from data gathered from 3 independent repetitions. (**c,d**) Columns represent a mean value from 12 ELISA readouts (*n* = 3 independent repetitions). Relative absorbance was normalized to values obtained for the control samples. Statistical significance: *ns* not statistically significant, *p* > 0.05, *p < 0.05, ***p < 0.001.

We started with the assessments of metabolic activity (using MTS assay; reduction of tetrazolium; impaired NAD(P)H metabolism^34^) and cell viability (using LDH assay; lactate dehydrogenase release to culture media, membrane damage^35^) that were applied to samples collected directly after OGD and after 24 h of reoxygenation. The results show that cell metabolism and viability depended on OGD duration (Fig. 2). Moreover, the induced changes are still present in cells after reoxygenation.

We tested how 1 h, 3 h, and 12 h of OGD affect the cell metabolism of SH-SY5Y cells (Fig. 2a,b). The MTS tetrazolium is reduced by cells to formazan soluble in the culture medium. The process is related to an NADH- and NADPH-dependent activity; thus, we assumed that the metabolic activity could be traced indirectly^34,36^. A lower absorbance of OGD cells compared to control could denote the lower metabolic activity of these cells. Our results show a significant reduction in formazan conversion after 3 h (7.1%, *p* < 0.001) and 12 h (41.6%, *p* < 0.001). After one hour of the cell exposure to OGD, cell viability (indirectly, the metabolic activity of cells) was similar to that of control cells, and no significant difference was identified (*p* = 0.262; Fig. 2a). In parallel, we checked membrane integrity by LDH assay related to the number of viable cells. A drop of about 13% – 17% was observed for cells after OGD (Fig. 2c, p < 0.001, Kruskal–Wallis ANOWA).

We expect that cells damaged by OGD will recover their ability to proliferate^37^. Therefore, MTS and LDH assays have also been applied to cells cultured in reoxygenation conditions. MTS results revealed significant changes in all groups of cells (Fig. 2b,d). After 24 h RO, we observed a drop in cell viability for all three groups of cells subjected to OGD, which dropped by 4.8% (*p* = 0.038), 13.3% (*p* < 0.001), and 20.1% (*p* < 0.001) after 3 h and 12 h of cell exposure to OGD, respectively (Fig. 2b). In LDH assay, a drop in the number of viable cells (Fig. 2d) was similar to cells measured directly after OGD (Fig. 2c, *p *< 0.001, Kruskal–Wallis ANOWA).

Comparison of the results from MTS and LDH assays shows that only metabolic activity was affected after prolonged OGD. It was not related to the number of viable cells (or, more precisely, to the impaired membrane integrity) but to altered metabolic activity.

### The effect of different OGD duration on mechanical properties of SH-SY5Y cells

**Figure 3:**
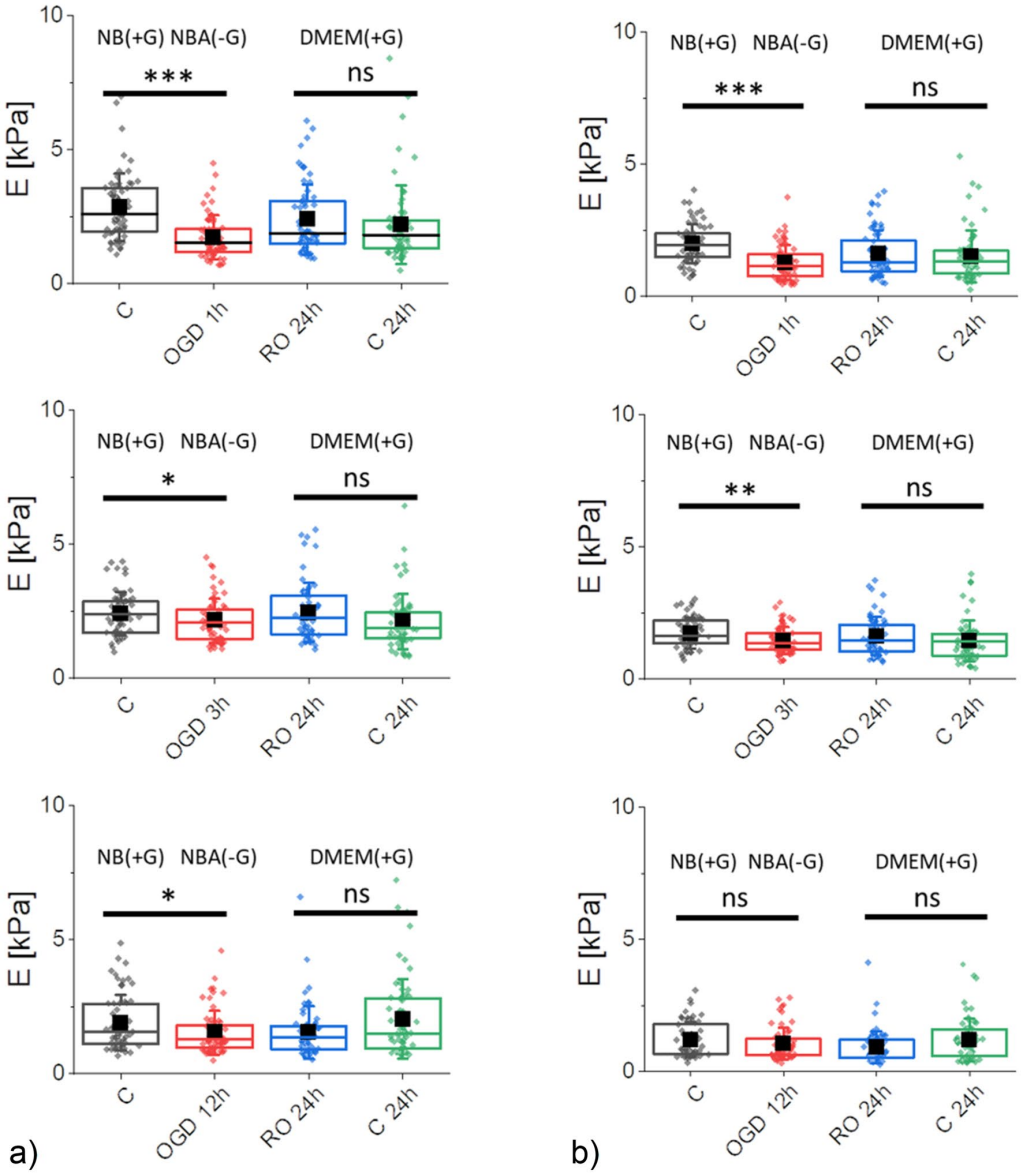
Nanomechanical properties of SH-SY5Y neuroblastoma cells after OGD treatment, quantified by the apparent Young's modulus calculated for the indentation depth of 400 nm **(a)** and 1200 nm **(b)**. Four groups of cells were compared: control (C, NB(+ G)), OGD cells (OGD 1 h, 3 h, or 12 h, NBA(−G)), re-oxygenated OGD cells (RO 24 h, DMEM(+ G)), and control, non-OGD cells (C 24 h) kept in DMEM(+ G) for the same time as re-oxygenated OGD cells. Box plots represent a median (black line), a mean (solid square), standard deviation (whiskers), and 25% and 75% percentiles (box) from *n* = 60 cells. Statistical significance: *ns* not statistically significant (*p *> 0.05), *p < 0.05, **p < 0.01, ***p < 0.001.

To assess whether the altered metabolic activity is related to the nanomechanical properties of SH-SY5Y cells, AFM working in a force spectroscopy mode was employed to conduct the measurements over a nuclear region of the cell (to avoid the influence of stiff substrates^38^). The nanomechanical properties were quantified by Young's (elastic) modulus calculated by applying Hertz-Sneddon contact mechanics^22,39^, assuming that a cone can approximate the shape of the probing pyramidal tip (Fig. 3).

Young's modulus was calculated at the shallow and deep indentations, i.e., 400 nm and 1200 nm, respectively. Alongside the already published data^22,33,40,41^, the nanomechanical response of cells measured at shallow indentation (400 nm) reflects mainly the mechanics of the actin cortex. Here, alterations in cell mechanics can be related to the remodelling of actin filaments underlying beneath the cell membrane. AFM can measure only cells attached to the underlying surface; thus, to a certain extent, the mechanical properties of cells reflect the mechanics of cells resistant to unfavorable conditions. Cells heavily affected by OGD detached from the surface therefore, they were not accessible for the AFM measurements. In our study, the mean of Young's modulus of OGD-treated cells, still attached to the surface, significantly dropped by about 39.2% (*p* < 0.001), 10.7% (*p* = 0.045), and 19.4% (*p* = 0.042) for 1 h, 3 h, and 12 h OGD in relation to control cells, respectively (Fig. 3a). Cells, kept in RO conditions, recover the nanomechanical properties of cells close to values obtained for control, non-OGD cells kept in DMEM(+ G) for 24 h (same duration as reoxygenation). The elastic moduli are 2.40 ± 1.31 kPa versus 2.22 ± 1.46 kPa (*p* = 0.401), 2.46 ± 1.09 kPa versus 2.12 ± 1.03 kPa (*p* = 0.084), and 1.55 ± 0.98 kPa versus 1.05 ± 1.48 kPa (*p* = 0.110) for OGD-treated and non-treated cells, correspondingly. Thus, we can conclude that the recovery of the actin cortex occurs independently of the OGD duration. The largest changes were observed in cells after 1 h OGD, but 24 h of reoxygenation simultaneously allowed cells to recover their mechanics almost fully. Longer OGD (3 h and 12 h) resulted in smaller mechanical changes than those observed after 1 h of OGD.

The analysis of deeper indentations (like here 1200 nm) can evaluate the combined contributions of the actin cytoskeleton and other structural components of cells such as microtubules or cell nuclei. Mechanics of cells measured directly after OGD shows a significant drop after 1 h and 3 h. The apparent Young's modulus dropped by 35.5% (*p* > 0.001) and 16.8% (*p* = 0.007), respectively (Fig. 3b). The OGD-induced mechanical changes were statistically insignificant after 12 h of cell exposure to such conditions (*p* = 0.188). These reductions in Young's modulus were similar to that observed for the 400 nm indentation. In AFM, the probing tip meets several structures during indentation. The first one is the cell membrane surrounding the cells. Due to its thickness, i.e., 10–20 nm, the mechanical response of only the cell membrane cannot be detected by AFM due to large errors in estimating the contact point between the probe and cell surface. Then, the actin cortex is mainly probed up to an indentation of 500 nm^42–44^. Such results suggest a weaker contribution of other cellular structures in the mechanical properties of SH-SY5Y cells. Structures such as the cell nucleus can also contribute to the mechanics of cells at larger indentations. But, the mechanical response is always burdened (or even hidden) by the mechanical response originating from the actin cortex. Therefore, only when AFM detects large changes in cell mechanics, the deeper cellular structures contribute to the mechanical properties of cells. A comparison of results obtained directly after OGD and after 24 h RO for analogous groups of cells shows that changes in mechanical properties were statistically insignificant, showing a lack of mechanical contributions from deeper cellular layers. These results demonstrate that the mechanical response mainly contains the dominant contribution from the actin cytoskeleton.

### Organization of actin cytoskeleton in OGD-treated SH-SY5Y cells

**Figure 4:**
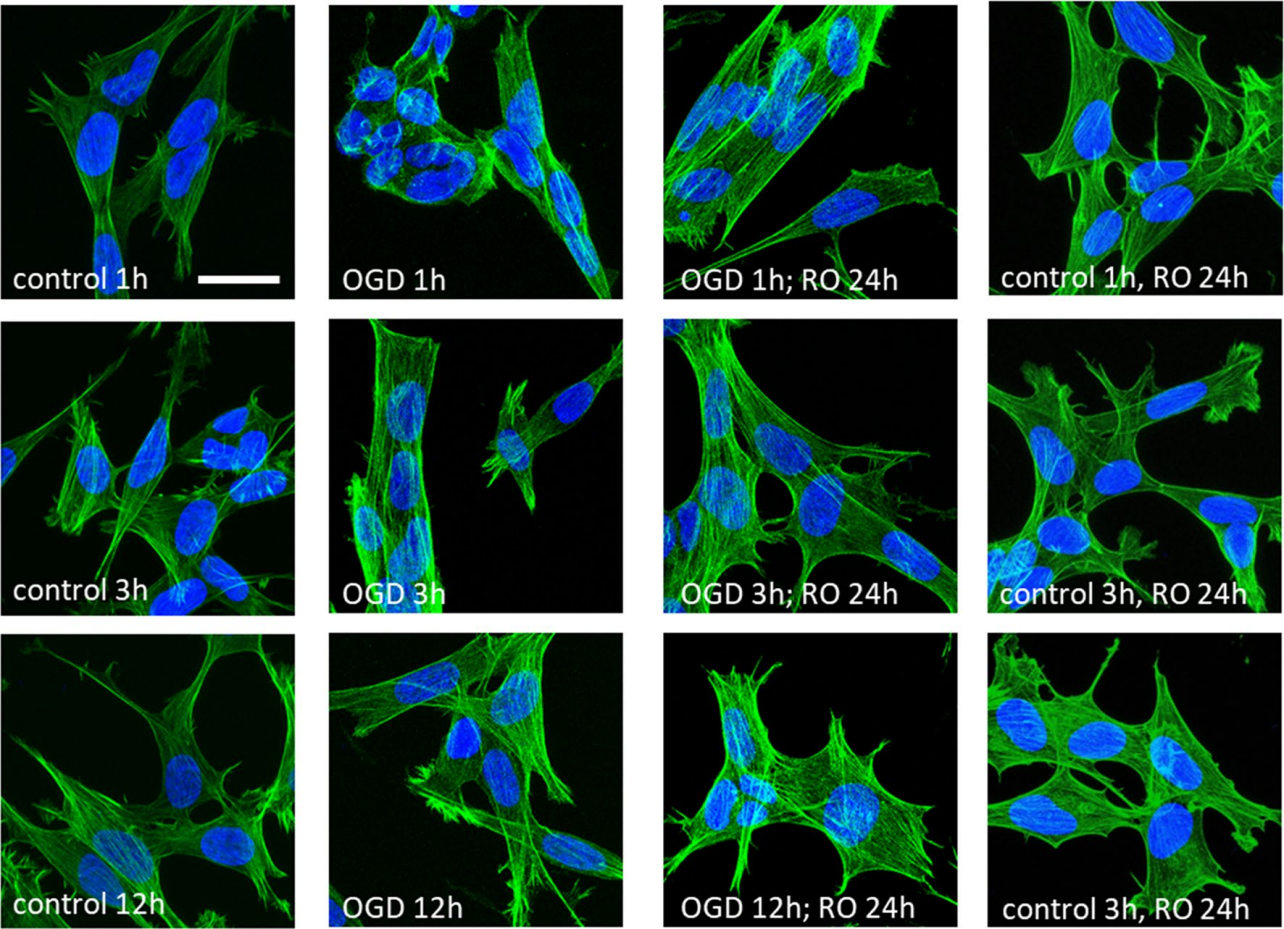
Confocal images of the actin cytoskeleton in OGD-treated and re-oxygenated cells. Staining: actin filaments—phalloidin conjugated with Alexa Fluor 488, cell nuclei—Hoechst 33342; scale bar 25 µm.

Phase-contrast images collected prior to the AFM measurements did not show any particular changes in the macroscopic morphology (Fig. 1b). OGD-treated cells reveal similar spindle and neuron-like morphology as control, non-treated cells, regardless of the OGD duration. As changes in cell mechanics are typically related to the organization of actin filaments, the confocal images with fluorescently labeled F-actin and cell nuclei were analyzed (Fig. 4).

In control and OGD treated cells, the actin cytoskeleton organization was very similar, showing nicely actin bundles spanning over the whole cell. The only exception was cells visualized directly after 1 h OGD, where cells change their morphology from a widely spread to a packed one (Fig. 4). This is consistent with the mechanical results showing the largest drop in the apparent Young's (elastic) modulus. The organization of actin filaments in cells undergoing longer OGD treatment (3 h and 12 h) was indistinguishable from control cells, supporting weak changes in nanomechanical properties.

### Cell spreading as a measure of cell attachment

**Figure 5:**
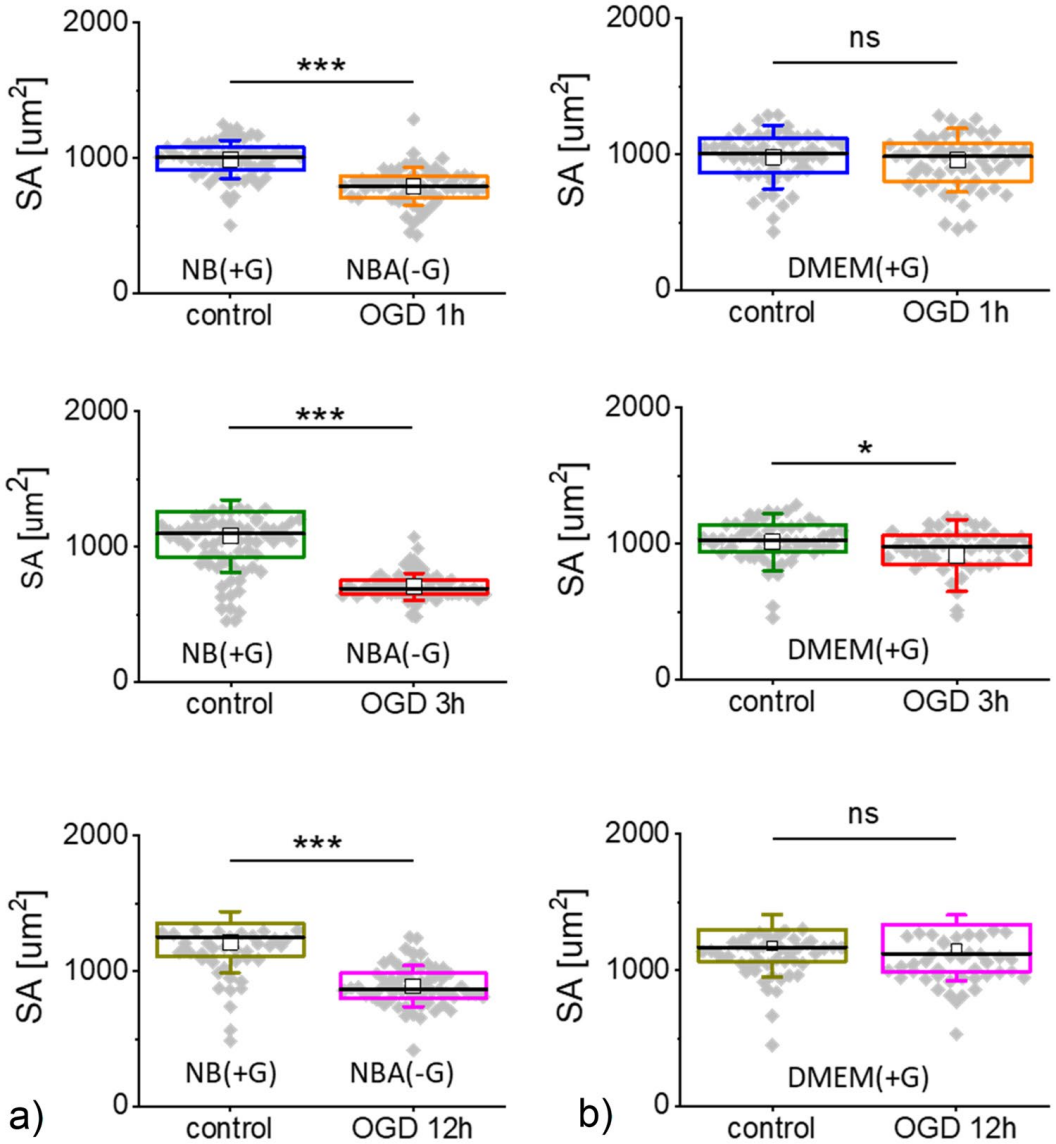
Spreading area (SA) of cells after OGD (**a**) and reoxygenation (**b**). Each dot denotes an average surface area of individual cells. Boxplot represents basic statistical parameters (mean, median, standard deviation, and 25% and 75% percentiles from *n* = 60 fluorescent images). Statistical significance: *ns* not statistically significant (*p* > 0.05), *p < 0.05, ***p < 0.001.

In our further steps, we performed a deeper analysis of the shape of individual cells using images recorded by epi-fluorescent microscopy (Fig. 5). For each group of cells, 60 images were analyzed (20 images per repetition).

The results revealed that OGD-treated cells have different surface areas indicating impairments in their spreading on the surface (Fig. 5a). The smaller the surface area, the worse their attachments/adhesion to the surface is. The weak attachment of cells to the underlying surface was recovered after allowing cells to grow in reoxygenation conditions (Fig. 5b). The largest change in spreading area was observed for OGD-treated cells, while during reoxygenation, cells return to the surface area of control, non-treated cells. These results indicate that the spreading of cells involves the remodelling of actin filaments, which in our case is strongly related to the OGD treatment of SH-SY5Y cells.

### OGD-related changes in the nucleus-to-cytoplasm ratio

**Figure 6:**
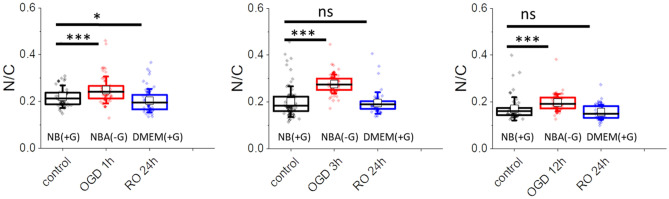
Nucleus to cytoplasm (*N/C*) ratio of cells after OGD treatments and reoxygenation. Each dot denotes an average value of individual cells. Boxplot represents basic statistical parameters, i.e., mean (open square), median (line), and standard deviation, from *n* = 60 cells. Statistical significance: *ns* not statistically significant *p < 0.05, ***p < 0.001.

Changes in cell surface area and lack of strong reorganization of the actin filaments suggest different mechanisms inducing the alterations in the nanomechanical properties of OGD-treated cells, such as changes in cell volume or a ratio between cytoplasm and nucleus. A ratio between surface areas occupied by cytoplasm (*C*) and cell nucleus (*N*) can quantify the latter (Fig. 6). The *N/C* value close to 1 indicates the dominant contribution of a cell nucleus in the surface area value, while its value close to 0 indicates a significant contribution from the cytoplasm.

The results show that the *N/C* ratio increases in cells visualized directly after OGD for all three tested timepoints and reaches the level of control cells after 24 h of cell reoxygenation in all groups. To verify whether a smaller spreading area and larger N/C ratio are correlated with the cell height, a cell height from the cross-section of confocal images (from Fig. 4) was estimated. The height of the cell in the central area was 7.3 µm ± 1.4 µm (*n* = 14 cells), 8.3 µm ± 2.0 µm (*n* = 11) 10.9 µm ± 2.8 µm (*n* = 10) for cells after 1 h, 3 h, and 12 h OGD, respectively. The lower *N/C* ratio did not correlate with the cell height increase but it correlated with single cell surface area. Its increase is related to the lower surface area of the cytoplasm showing changes in the cell cytoskeleton organization.

### Cofilin level in OGD-treated SH-SY5Y cells

**Figure 7:**
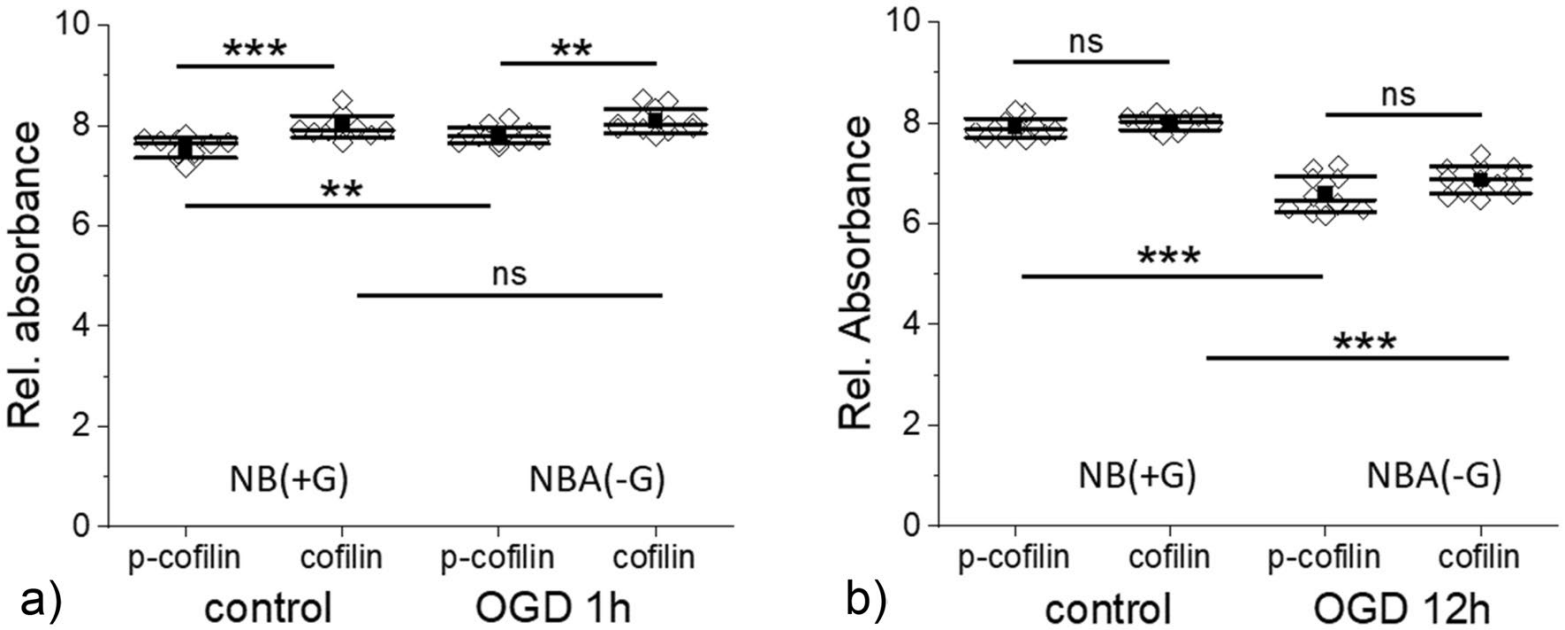
Cofilin and p-cofilin (phosphorylated cofilin) expression level in SH-SY5Y cells upon 1 h **(a)** and 12 h **(b)** OGD. Control cells were kept in NB(+ G), while OGD cells were kept in NBA(−G). A mean (black square), median (black middle line), standard deviation (SD, outside black lines) were determined from data gathered from 3 independent repetitions. Statistical significance (*ns* not statistically significant, **p < 0.01, ***p < 0.001).

Cofilin is an actin-regulating protein that quickly responds to various cellular processes. Alterations in calcium ions, reactive oxygen species, ATP, or pH will result in quick dephosphorylation (activation) of cofilin^45^. Cofilin severs actin filaments but it can also promote actin filament disassembly^46^. Instead, it creates new nucleation centers allowing for quick branching, polymerization, and depolymerization in a concentration-dependent manner^47,48^. The results of cofilin and p-cofilin (phosphorylated cofilin) expression levels in control and OGD-treated cells are presented in Fig. 7.

We observed the difference in the cofilin and p-cofilin levels in control cells that could be linked with a lower glucose level observed in proliferating cells in a given medium volume during a certain culture time^40^. When cofilin/p-cofilin was assessed in control cells simultaneously as cells after 1 h of OGD, the concentration of cofilin was about 5% higher than p-cofilin (*p* < 0.001). In 1 h OGD treated cells, the concentration of cofilin was only 3.5% larger than p-cofilin (*p* < 0.002). In cells exposed to longer OGD duration, the expression level of cofilin in relation to p-cofilin vanishes (the same protein level was observed in cells after 3 h and 12 h OGD (Fig. 7b shows results of cofilin/p-cofilin expression in SH-SY5Y cells after 12 h OGD). Next, we compare the differences between the expression level of cofilin (or p-cofilin) for control and OGD-treated cells. The determined *p*-values were (i)* p* = 0.006 (p-cofilin, between control and OGD (1 h) cells) and *p* = 0.198 (cofilin, between control and OGD (1 h) cells); (ii)* p* < 0.0001 (between control and OGD (3 h) cells, regardless of the cofilin status); (iii)* p* < *0.0001* (between control and OGD (3 h) cells, regardless of the cofilin status). These results show that the ratio between cofilin/p-cofilin changes significantly during 1 h OGD and vanishes for a longer duration of OGD. Interestingly, the expression level of cofilin and p-cofilin in OGD cells decreases with OGD duration. Only the expression level of p-cofilin changed.

## Discussion

Oxygen and glucose deprivation (OGD) is commonly used to study cerebral ischemic stroke. It mimics the process of a sudden disruption of blood flow to the brain. The lack of blood supply leads to decreased oxygen and glucose levels in the brain^49^. The induced injuries activate various biochemical processes such as perturbation of calcium homeostasis^50^, malfunction of endoplasmic reticulum and mitochondria^51^, and increased oxidative stress linked with DNA damage^52^. They directly affect cell morphology, which suggests changes in the mechanical properties of OGD-treated cells. In our work, AFM was applied to probe nanomechanical properties at the cellular level. Nanomechanics has already been reported to be altered during stroke^53^. The results have shown that tissue mechanics changes within the region affected by stroke and, also, at a distance from the stroke site^53^. AFM has shown the altered mechanical properties of the brain region severely affected by ischemia. Neuronal cells are mechanosensitive and highly responsive to altered mechanics of the surrounding environment^54^. Thus, changes in the mechanical properties of ischemic tissue denote also changes in the functioning of neuronal cells.

Our study focused on the nanomechanical properties of SH-SY5Y cells subjected to OGD of different duration followed by 24 h reoxygenation. AFM-based elasticity measurements were conducted at the shallow and deep indentations, which enabled us to quantify the changes occurring mainly in the network of actin filaments. A drop of Young's modulus, a measure of cell deformability^22^, observed in cells subjected to OGD, suggests the reorganization of the cell cytoskeleton at the layer composed of the actin filaments. The most significant drop in Young's modulus was observed in SH-SY5Y cells measured directly after OGD. When cells were allowed to grow in fully re-oxygenated conditions, their elastic properties returned to the level of control cells. The cell metabolic activity (MTS assay) and cell viability (LDH assay) showed that the number of alive cells remained within 83–88% for both control and OGD-treated cells. However, the metabolic activity of cells decreases with OGD duration, oppositely to changes observed in nanomechanical measurements.

Based on the obtained results, we propose the following mechanism leading to cell deformability changes during OGD. The observed time-dependent decrease of Young's modulus in control, non-OGD treated SH-SY5Y cells was gradual regardless of the indentation depths chosen for the analysis (for low indentations of 400 nm and deeper indentations of 1200 nm). Probably, it reflects the impact of glucose consumption on the mechanical properties of cells. As the dynamic of assembly and disassembly of F-actin is strongly dependent on the accessibility of adenosine triphosphate (ATP) molecules (ATP- G-actin binds to barbed end three times faster than GTP-G-actin^55^), the reduction of ATP resulted in slow disassembly of cytoskeleton resulting in a gradual decrease of Young's modulus. A significantly lower level of ATP was reported to lead to cell softening^56^.

Several actin-associated proteins regulate actin polymerization/depolymerization as dynamic assembly and disassembly of the actin cytoskeleton are required for many biological processes, such as cell division, cell motility, endocytosis, and morphogenesis. These actin regulatory proteins contribute to nucleation, depolymerization, and fragmentation when a reorganization of the actin filaments is needed^57–60^. Cofilin is one of such proteins. It disassembles F-actin into tiny fragments of fibrous actin^61^. Although severing of F-actin by cofilin is independent of energy addition, the extensive reorganization of actin filaments demands high energy supplies^62^, in particular, to reach the level detected by AFM. It seems to be supported by the MTS assay showing unaltered metabolic activity of OGD-treated cells after one hour of the treatment. We postulate that in our measurements during the initial OGD, alterations in the mechanical properties of cells reflect the disassembling effect of cofilin on the cortical actin. The effect is evident in cells after 1 h of OGD. It is additionally detectable in the experiments, in which changes in the effective surface area of a single cell are quantified. The OGD duration of 1 h is sufficient to induce a significant (~ 20%) reduction of the mean single cell surface area and increase the cell height. Such an increase suggested a more sparse actin scaffold and softening of the cells.

For longer OGD (3 h and 12 h), the cofilin-induced actin polymerization seems to be attenuated as changes in the nanomechanical properties of OGD-treated cells are less pronounced. Severing and depolymerization of actin filaments by cofilin create many small F-actin fragments with new barbed ends needed for their polymerisation^63,64^. Simultaneously, after prolonged OGD time, cells are metabolically impaired, as the MTS assay reveals a significant drop in the number of metabolically active cells. It makes the reorganization of the actin cortex less favorable, indicating that cofilin activity is inhibited by phosphorylation of the serine residues at position 3 near the N-end for a longer OGD duration. As a result, polymerization and stabilization of actin filaments are observed^65,66^. Such an effect leads to decreased cell deformability (cells become more rigid as Young's modulus increases) in cells with low metabolic activity. The latter affect the rebuilding of the actin filaments network, which is low, as shown by small values of the surface area of a single cell in cells after 3 h and 12 h of OGD.

After reoxygenation, the surface area went back to the levels of controls, but mechanical properties did not fully recover. When comparing short-time OGD, the elasticity of the cortical layer (400 nm) was well restored. The deep indentation, however, shows irreversible changes. Both shallow and deep indentations were irreversible after 12 h OGD. These results contradict quantitative observations for actin and tubulin networks in fluorescence microscopy, where any significant change cannot be clearly observed. It indicates that changes in cell mechanics are more complex and cannot be explained only by actin (de) polymerization. Actin rearrangement via activation of profilin, cofilin, and gelsolin, phosphorylation of myosin light chain, and changes in membrane spectrin cytoskeleton might be involved^67^. It should also be noted that in most studies, OGD constitutes the oxygen and glucose deprivations considered in parallel. Separating the specific oxygen- and glucose-related contributions calls for experiments conducted in conditions of either glucose or oxygen depletion^68^. The AFM-derived nanomechanical properties of OGD and non-OGD treated cells reveal a dominant role of glucose deprivation in 12 h of OGD conditions that hinders the oxygen depletion effect.

Numerous research that use the OGD model to understand mechanisms involved in brain impairments^68,69^ has demonstrated that the pathological process of ischemic stroke involves complex mechanisms acting on various cell types. These studies focus on cell or molecular biology aspects. Cofilin, being involved in the dynamic turnover of actin filaments, affects membrane integrity, receptor transport, and signal transduction. Understanding mechanisms responsible for cofilin-related changes of cytoskeleton remodeling, promise a potential way to inhibit cofilin activity that might induce neuroprotection through targeting diverse cellular components and multiple pathways^70,71^.

## Methods

### Cell culture

For experimental procedures, an undifferentiated SH-SY5Y human neuroblastoma cell line was used. Cells were cultured in a Dulbecco's Modified Eagles' Medium (DMEM, ATCC, LGC Standards) supplemented with 10% Fetal Bovine Serum (FBS, ATCC, LGC Standards). Cells were cultured in 35 cm^2^ culture flasks (TPP) and passaged (< 10) into the corresponding plastic media required in each experiment. Cells were cultured in the CO_2_ incubator (NUAIRE) at 37 °C and 5%CO_2_/95% air atmosphere.

### OGD experiments

Cells were passaged from the culture flask to the Petri dish (TPP) and kept in the CO_2_ (37 °C and 5%CO_2_/95% air atmosphere) for 24 h in the DMEM (ATCC, LGC Standards) supplemented with 10% FBS. DMEM contains 4500 mg/L glucose and 1 mM sodium pyruvate. After this time, the medium was replaced either with (1) neurobasal medium (NB) containing glucose (control cells, undergoing the same treatment as OGD cells but without applying OGD conditions) or with neurobasal A medium (NBA, without glucose used to create OGD conditions). NB is optimized for prenatal and fetal neurons. NBA is optimized for growing postnatal and adult brain neurons. These two media differ only in osmolality (260 mOsm versus 235 mOsm, respectively).

OGD conditions were obtained in the following way. Cells were placed in the temperature-controlled table CO_2_ incubator (Olympus) at 37 °C. The incubator was connected to a gas exchange 3-input system (Tokai Hit) supplying air, N_2,_ and CO_2_. In our system, CO_2_ concentration remained constant (5%) while the air was replaced by N_2_, resulting in an oxygen concentration of 0.1%. The oxygen level was maintained constant by applying a gas flow at a level of 150 ml/min. These parameters were kept constant for 1, 3, and 12 h. Immediately after applying OGD, cells were analyzed using various techniques: MTS and LDH assays, atomic force microscopy (AFM), epifluorescence, and confocal microscopy.

### MTS assay

The viability (and indirect metabolic activity) of SH-5YSY cells were verified by using an MTS colorimetric test (Promega). Cells were cultured in 24-well plates in 1 ml of the culture medium (DMEM). Next, 100 µL of MTS reagent (tetrazolium compound) was added to the cells. Then, cells were incubated at 37 °C in 95% air/5% CO_2_ atmosphere in the CO_2_ incubator (Nuaire) for 2 h. The MTS method reduces tetrazolium compounds by viable cells to generate a colored formazan product soluble in cell culture media. The final volume of 1.1 mL was pipetted to 96-well plates with 100 µL per hole. The absorbance (OD = 490 nm) was recorded for 0 h, 3 h, and 24 h after OGD using a spectrophotometer (ELISA SPECTROstar Nano, BMG LABTECH). The MTS assay was repeated three times.

### LDH assay

The cytotoxic effect of OGD was quantified by using CyQUANT™ LDH Cytotoxicity Assay Kit (Invitrogen). Cells were plated on 24-well plates in 1 ml of the corresponding culture medium (Fig. 1). LDH level was evaluated in the following samples, i.e., (i) control and OGD cells, (ii) control and OGD cells treated with lysis buffer (10% by volume, 45 min in the CO_2_ incubator), (ii) culture medium (supernatant) taken from control or OGD cells and separately treated with lysis buffer in an analogous way as (ii) to verify how much cells detached during the medium exchange. Then, 50 µl of the medium from each sample type was aspirated from each well and transferred into a 96-well plate. Next, to each well, 50 µl of the reaction mixture was added. After 1 h of incubation in conditions protecting against light exposure, 50 µl of stop solution was added. Lactate dehydrogenase (LDH) is a cytosolic enzyme present in various cell types. Damage to the cell membrane results in a release of LDH to the surrounding medium, which can be quantified by using LDH as a catalytic enzyme. It converts lactate to pyruvate via NAD + reduction to NADH. Oxidation of NADH by diphosphorase reduces a tetrazolium salt to a red formazan product. OD at 490 nm was registered using an ELISA reader (Ledetect 96 ELISA, LED-based microplate reader, Labexim Products) to detect it. The level of formazan is directly proportional to the level of LDH in the surrounding medium. To obtain cell viability level the following equation was used:

1 $$\%\left(cell\, viability\right)=1-\frac{expLDH}{maxLDH}$$

The relative maximum percentage error was calculated using the error propagation method:

2 $$\%\left(cell \,viability\right)=\frac{1}{maxLDH }\left(\Delta \left(expLDH\right)+\frac{expLDH}{maxLDH }\Delta \left(maxLDH\right)\right)$$

where *expLDH* is the experimental LDH release (OD490), *maxLDH* is the maximum LDH release that is the sum of LDH release in lysis buffer by treated samples (cells and supernatants of control and OGD-treated cells, respectively).

### Phospho-cofilin/cofilin assay

To obtain changes in cofilin activity level, CytoGlow™ Cofilin (Phospho-Ser3) Colorimetric Cell-Based ELISA Kit was applied (Assay Biotechnology) to monitor target proteins concentration, here, in cells undergoing OGD treatment. Briefly, SH-SY5Y cells (50,000 per well) were plated on a 96-well plate. After OGD experiments, cells were fixed using 4% paraformaldehyde and washed three times with 200 µl with Wash Buffer (WB) for 5 min, each time gentle shook. Then, 100 µl of quenching solution was added for 20 min at room temperature (RT), followed by 3 times washing with WB for 5 min at a time. Next, 200 µl of Blocking Buffer was added for 1 h at RT, and, afterwards, the plate was washed again (3 × times, WB at RT). Then, a solution of 50 µl of each primary antibody against phosphorylated cofilin (Anti-Cofilin (Phospho-Ser3) antibody), cofilin (Anti-cofilin antibody) and Glyceraldehyde 3-phosphate dehydrogenase, GADPH (Anti-GAPDH antibody) was added to the corresponding well and incubated for 16 h (overnight) at 4 °C. Afterwards, they were rinsed 3 times with 200 µl of WB for 5 min. In the next step, secondary antibodies (horseradish peroxidase (HRP)-conjugated antiRabbit IgG antibody and/or HRP-conjugated anti-Mouse IgG antibody) were added (50 µl) for 1.5 incubation at RT. After incubation, the plate with cells was washed, and 50 µl of Ready-to-Use Substrate was added to each well for 30 min at RT, followed by adding a Stop Solution. OD at 450 nm was immediately read using a microplate reader (ELISA SPECTROstar Nano, BMG LABTECH).

### Atomic force microscope (AFM)

The mechanical properties of cells were measured using AFM (CellHesion, Bruker-JPK, Germany). The microscope is equipped with a constant temperature system. In our experiments, the temperature was set to 32 °C to provide the cell survival conditions and the cantilever stability. Cells were indented with silicon nitride cantilevers (ORC-8, Bruker) characterized by a nominal spring constant of 0.03 N/m and an open half-angle of 36°. All measurements were conducted in a force spectroscopy mode. The spring constants of used cantilevers were determined using the Sader method^72^. The average value was 0.058 ± 0.005 for *n* = 8 cantilevers. A force map of 6 per 6 pixels (corresponding to a 6 µm × 6 µm scan size) was recorded on each cell.

The force curves (i.e., the dependence of the cantilever deflection recorded as a relative sample position) were acquired at the approach/retract velocity of 8 µm/s. On individual plastic Petri dishes, two groups of force curves were collected. First, calibration curves were acquired on a Petri dish bottom surface (a reference calibration curve). Next, force curves were recorded on living cells. Next, force curves were recorded on living cells. Force curves were collected by setting a grid of 6 × 6 points that corresponded to 6 µm × 6 µm scan area. A grid was set over the nuclear region to minimize the influence of the underlying stiff plastic surface. All measurements were conducted in DMEM and were repeated three times.

### Young's modulus determination

The subtraction of the calibration curves from a curve collected on a living cell produces the relation between load force and indentations depth. This relation was analyzed using the Hertz-Sneddon contact mechanics. For the cone approximated the AFM probe, the relation between load force and indentation depth is following:

3 $$F\left(\delta \right)= \frac{2\cdot \mathrm{tan}(\alpha )}{\pi }\cdot \frac{{E}_{cell}}{1-{\mu }^{2}}\cdot {\delta }^{2}$$

where *F* is load force, *δ* is the indentation depth, Ecell is the apparent Young's modulus of the cell, and *µ* is the Poisson's ratio (equaled to 0.5 assuming that cells are incompressible materials). The final modulus value was expressed as a mean and standard deviation from all measured cells.

### Fluorescence (epifluorescence and confocal) microscopy

Visualization of actin filaments, microtubules, and cell nuclei was performed by using Olympus IX83 (Olympus, Japan) fluorescence microscope equipped in objectives 20× and 40× magnification, 100 W mercury lamp (illuminating the whole cell area uniformly), and a set of filters to record emission at 594 nm and 420 nm. Images were collected using Orca Spark digital camera providing a 2.3 megapixel (1920 × 1200) pixel image and analyzed with ImageJ (ImageJ 1.53e https://imagej.nih.gov/ij/). Cells cultured on 24-well plates were fixed in 3.7% paraformaldehyde, then they were washed with phosphate-buffered saline (PBS, Sigma), treated with a cold 0.2% Triton X-100 solution, and again washed with the PBS buffer. Afterwards, cells were incubated with β-tubulin antibody conjugated with Cy3 for 24 h. The next day, samples were stained with phalloidin conjugated with AlexaFluor 488 dye during 1 h incubation. Cell nuclei were stained by 10 min incubation with Hoechst 33342  dye.

Confocal images of actin and microtubular cytoskeleton were recorded at the Laboratory of in vivo and in vitro Imaging (Maj Institute of Pharmacology Polish Academy of Science, Cracow, Poland). They were recorded using a Leica TCS SP8 WLL confocal microscope equipped with new-generation HyD detectors set at 415–450 nm (Hoechst) and 509–560 nm (Alexa Fluor 488). Fluorescent dyes were excited by diode lasers: 405 nm (Hoechst) and white light laser with emission wavelength set at 499 nm (AlexaFluor 488). Images were registered using an oil immersion 63 × objective lens (HC PL APO CS2 NA 1.40).

### Surface area determination

A single cell effective surface area (SA) was applied to characterize how well cells spread on the surface at given conditions. This value describes the average surface area occupied by an individual cell. Images of fluorescently stained cells (F-actin using phalloidin-Alexa Fluor 488 dye, cell nuclei by Hoechst 33342) were binarised using ImageJ software. From these images, the surface area occupied by cells was determined. Next, cell nuclei were manually counted to receive the number of cells. Finally, the surface area occupied by cells was divided by the number of cells enabling the calculation of the effective surface area of a single cell. Images were acquired during three repetitive experiments, which resulted, in total, in 60 values of the effective surface area of a single cell per condition to be analyzed. The total number of cells was at least 8000 cells.

### Nucleus- to -cytoplasm (N/C ratio)

To obtain the N/C, the effective area of individual cell nuclei was quantified analogously as the effective surface area of a single cell was determined. Next, the effective surface area of a single nucleus was divided by the effective surface area of a single cell. The total number of images analyzed was 60 per condition (20 per repetition).

### Statistical analysis

All data are presented as the mean ± standard deviation from *n* repetitions. In all figures, box plots were applied to show the basic statistical descriptors: mean (open square), median (black line), standard deviation (whiskers), and 25% and 75% percentiles (box). Statistical significance was verified by applying the non-parametric Mann–Whitney test (Origin 9.2 Pro). Significance is indicated by *p* values (*ns* – not statistically different, *p* > 0.05; *p < 0.05, **p < 0.01, ***p < 0.001).

## Data Availability

Correspondence and requests for materials should be addressed to ML.
